# Integrative biomarker and drug target discovery in osteosarcoma: traditional experimental approaches and AI-enabled insights

**DOI:** 10.3389/fphar.2026.1791228

**Published:** 2026-04-07

**Authors:** Zhihao Gao, Chongqin Wu

**Affiliations:** 1 Department of Laboratory Medicine, Chong Gang General Hospital, Chongqing, China; 2 Department of Orthopedics, Chong Gang General Hospital, Chongqing, China

**Keywords:** artificial intelligence, biomarkers, drug sensitivity prediction, multi-omics integration, osteosarcoma, precision pharmacology, therapeutic targets

## Abstract

Osteosarcoma is the most common primary malignant bone tumor and remains a major clinical challenge due to frequent metastasis, chemoresistance, and pronounced molecular heterogeneity. Despite substantial advances in understanding disease biology, clinically actionable biomarkers and therapeutic targets that can reliably support precision treatment decisions remain limited. Traditional experimental approaches have yielded important mechanistic insights into osteosarcoma pathogenesis, but their hypothesis-driven nature and limited scalability constrain the ability to capture complex regulatory interactions. Recent progress in high-throughput sequencing and multi-omics profiling, together with advances in artificial intelligence (AI), has enabled more systematic interrogation of high-dimensional molecular landscapes. By integrating heterogeneous datasets, AI-based analytical frameworks can identify composite biomarker patterns, regulatory hubs, and candidate druggable vulnerabilities that better reflect tumor complexity and treatment heterogeneity. In parallel, computational strategies for drug sensitivity prediction and drug repurposing are emerging as complementary tools for accelerating therapeutic hypothesis generation and prioritizing candidate interventions in osteosarcoma. In this mini-review, we summarize recent progress in biomarker discovery and therapeutic target identification, with an emphasis on how traditional experimental evidence and AI-driven analyses function as complementary components within an integrated discovery-to-validation framework. We discuss key challenges in translational validation and highlight future directions for integrating data-driven discovery with pharmacological and clinical research to advance precision therapy for osteosarcoma.

## 1 Introduction

Osteosarcoma is the most common primary malignant bone tumor and predominantly affects children, adolescents, and young adults (Sheng et al., 2021). Despite advances in surgical techniques and multi-agent chemotherapy, long-term survival has largely plateaued over the past several decades (Yu and Yao, 2024). While patients with localized disease can often be managed with limb-salvage surgery combined with neoadjuvant and adjuvant chemotherapy, the prognosis remains poor for those presenting with metastasis or developing recurrence (Yang et al., 2018; He et al., 2017). Pulmonary metastasis, chemoresistance, and pronounced inter- and intra-tumoral heterogeneity remain the principal clinical challenges limiting therapeutic efficacy (Yang et al., 2020; Zhang et al., 2018; Wen et al., 2022).

In current practice, clinical decision-making in osteosarcoma still relies mainly on imaging, histopathology, and a small set of clinicopathological variables such as tumor size, anatomical site, and histologic response to preoperative chemotherapy (El Motassime et al., 2025). However, these conventional indicators do not provide sufficient sensitivity or specificity for robust stratification of prognosis or treatment response (Xin and Wei, 2020). Consequently, some patients are exposed to intensive treatment regimens without clear benefit, whereas others who may require alternative or intensified therapeutic strategies are not identified in a timely manner (Zhu et al., 2023). These gaps highlight an urgent need for reliable biomarkers that can support early detection, risk stratification, treatment selection, and longitudinal monitoring.

In parallel, the identification of effective therapeutic targets in osteosarcoma remains challenging (Tarone et al., 2025). Although numerous oncogenic pathways and molecular drivers have been reported, their translation into clinically effective targeted therapies has been limited (Hu et al., 2025; Yan et al., 2024). This difficulty largely reflects the biological complexity of osteosarcoma, which is characterized by extensive genomic instability, molecular heterogeneity, and dynamic tumor microenvironmental interactions.

Recent advances in high-throughput sequencing, multi-omics profiling, and computational biology have expanded the ability to interrogate osteosarcoma at scale and across molecular layers (Tang et al., 2023; Jia et al., 2023). Within this landscape, artificial intelligence (AI)–based approaches are increasingly used to integrate heterogeneous datasets, learn informative representations, and prioritize candidate biomarkers and therapeutic targets for downstream testing. In this review, “integration” refers to an operational workflow that links data-driven prioritization to experimental validation: AI-guided analyses help narrow and organize candidates, while traditional experiments provide mechanistic support and functional confirmation, forming an iterative discovery-to-validation loop rather than two parallel lines of evidence (Jiang et al., 2024; Yuan et al., 2023; Sarvepalli and Vadarevu, 2025; Katsianou et al., 2025).

Accordingly, this mini review is positioned as a methodological integration framework, using selected osteosarcoma examples to illustrate how evidence is generated and validated rather than attempting a comprehensive catalog of all reported biomarkers and targets. We discuss their respective strengths and limitations, highlight key challenges in translational validation, and outline future directions toward more precise and mechanism-guided therapeutic approaches.

## 2 Conventional biomarker discovery in osteosarcoma

### 2.1 Tissue-based biomarkers and histopathological indicators

Tissue-based biomarker identification has long been a central focus of osteosarcoma research, aiming to improve diagnostic accuracy and prognostic stratification (Reddy et al., 2026). Immunohistochemistry and transcriptomic analyses are commonly applied to tumor specimens to evaluate gene and protein expression patterns associated with tumor aggressiveness, metastatic potential, and clinical outcomes (Liu F. et al., 2025). Multiple candidate markers have been reported to correlate with histological grade, tumor necrosis rate following neoadjuvant chemotherapy, and risk of recurrence, highlighting their potential clinical relevance (Huang G. et al., 2024). However, the clinical utility of tissue-derived biomarkers remains constrained. Pronounced intra- and inter-tumoral heterogeneity can make single biopsy specimens non-representative in clinical sampling. Variability in assay protocols and scoring systems further reduces reproducibility across cohorts, limiting generalizability and routine implementation of tissue-based biomarkers.

### 2.2 Circulating biomarkers and liquid biopsy approaches

Circulating biomarkers have emerged as attractive, minimally invasive tools for osteosarcoma monitoring (Maqueda et al., 2024). Serum proteins, circulating tumor DNA (ctDNA), microRNAs, and extracellular vesicles have been investigated for their potential roles in early diagnosis, prognostic assessment, and treatment response evaluation (Huber et al., 2023; Smolle et al., 2025). Compared with tissue-based analyses, liquid biopsy approaches enable longitudinal sampling and may facilitate earlier detection of disease progression or recurrence (Barris et al., 2018). Nevertheless, translation into clinical practice remains limited. Reported results are frequently inconsistent, largely due to differences in sample processing, detection platforms, and analytical strategies. In addition, limited sensitivity in early-stage disease and small cohort sizes have impeded robust validation, restricting most circulating biomarkers to exploratory research settings.

### 2.3 Omics-based biomarker discovery and its limitations

High-throughput omics technologies, including bulk RNA sequencing, proteomics, and metabolomics, have substantially expanded the scope of biomarker discovery in osteosarcoma (Zhang Z. et al., 2025). These approaches enable systematic profiling of molecular alterations and have identified dysregulated pathways involved in proliferation, cell death, metabolism, and immune regulation (Zhang et al., 2021). Omics-derived signatures offer valuable insights into disease biology and hold promise for improving prognostic modeling (Zhang et al., 2022; Wu et al., 2023). However, their translational impact remains limited. Many studies rely on small cohorts without independent validation, and single-omics analyses often fail to capture cross-layer regulatory interactions. Moreover, the high dimensionality of omics data increases the risk of overfitting when conventional analytical methods are applied without appropriate integration strategies.

Collectively, conventional biomarker discovery approaches have established an important foundation for understanding osteosarcoma biology but remain insufficient to fully address the disease’s molecular complexity and clinical heterogeneity. These limitations highlight the need for integrative, data-driven strategies capable of prioritizing clinically relevant biomarkers, thereby motivating the incorporation of artificial intelligence–based methodologies discussed in subsequent sections.

## 3 Traditional identification of therapeutic targets and mechanistic insights

### 3.1 Oncogenic signaling pathways as therapeutic targets

Traditional studies have identified multiple dysregulated oncogenic signaling pathways that contribute to osteosarcoma initiation, proliferation, invasion, and metastasis (Jeddo et al., 2020; Li et al., 2025; Meuten et al., 2025). Aberrant activation of pathways governing cell growth, survival, and differentiation is frequently observed and is associated with aggressive tumor behavior and resistance to conventional chemotherapy (Huang X. et al., 2024; Liao et al., 2025). Experimental perturbation of these pathways affects proliferation, apoptosis, and migration, supporting their therapeutic relevance (Liang et al., 2022; Cheng et al., 2016). However, clinical translation of single-pathway–targeted strategies has been limited. Pathway redundancy and compensatory signaling can blunt efficacy, allowing malignant phenotypes to persist despite pathway inhibition.

### 3.2 Transcriptional regulation, epigenetic modulation, and metabolic reprogramming

Beyond classical signaling cascades, transcriptional and epigenetic dysregulation plays a critical role in osteosarcoma progression (Walker et al., 2025). Alterations in transcription factors and chromatin-modifying regulators contribute to tumor cell plasticity, stemness, and adaptive responses under therapeutic pressure (Perkins et al., 2024; Zhang et al., 2023; Dos Santos et al., 2023). These programs intersect with metabolism, reshaping energy use and biosynthesis to support growth and survival. Metabolic reprogramming has emerged as an important feature of osteosarcoma biology, influencing both disease aggressiveness and therapeutic response (Shi et al., 2023; Yin et al., 2025). Although targeting metabolic dependencies represents a promising therapeutic avenue, pronounced metabolic heterogeneity and adaptive flexibility remain major barriers to effective clinical application.

### 3.3 Cell death pathways and therapy resistance

Dysregulation of programmed cell death is a central mechanism underlying chemoresistance and disease recurrence in osteosarcoma (He et al., 2014; Meng et al., 2020). Alterations in apoptotic signaling, along with disruption of non-apoptotic cell death processes, enable tumor cells to evade cytotoxic stress induced by chemotherapy (Tang et al., 2025; Xiao et al., 2018; Zhao et al., 2021). Experimental modulation of these pathways can partially restore drug sensitivity and suppress tumor growth in preclinical models (He et al., 2023; Chen et al., 2024). Nevertheless, cell death pathways are tightly interconnected with broader signaling and metabolic networks. Targeting single regulators is often insufficient, underscoring the need for strategies that account for network-level interactions.

### 3.4 Tumor microenvironment–associated targets and translational limitations

The osteosarcoma tumor microenvironment, composed of immune cells, stromal elements, and extracellular matrix components, plays a crucial role in tumor progression and therapeutic response (Cascini et al., 2021; Wang et al., 2025). Tumor–microenvironment interactions influence immune evasion, metastatic dissemination, and treatment sensitivity, and have therefore been explored as potential therapeutic targets (Orrapin et al., 2024; Wu et al., 2025; Tian et al., 2023). Despite encouraging mechanistic insights, translation of microenvironment-focused strategies into effective therapies remains challenging. The dynamic, context-dependent nature of tumor–host interactions and the constraints of available models and clinical specimens complicate target validation and clinical translation.

Overall, traditional approaches have yielded important mechanistic insights and led to the identification of numerous candidate therapeutic targets in osteosarcoma. However, their hypothesis-driven focus on single pathways or isolated mechanisms limits the ability to capture molecular heterogeneity and network-level complexity. These limitations highlight the need for complementary data-driven strategies to refine target prioritization and improve therapeutic precision, as elaborated in the following sections.

## 4 AI-driven multi-omics integration for biomarker discovery

### 4.1 Rationale for AI-Based integration in osteosarcoma research

**FIGURE 1:**
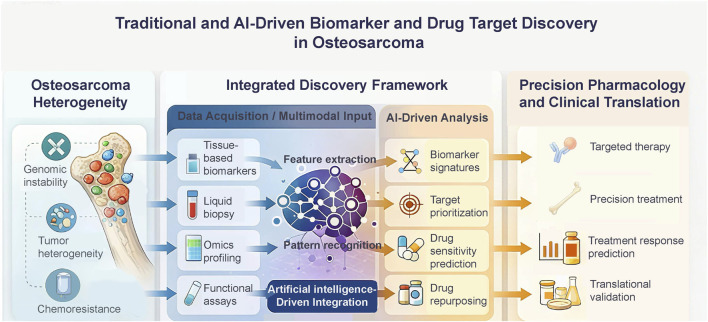
Integrative framework for biomarker and therapeutic target discovery in osteosarcoma. Integration of traditional experimental strategies with AI-driven multi-omics analysis enables biomarker identification, target prioritization, and drug sensitivity prediction for precision pharmacology.

The molecular landscape of osteosarcoma is characterized by extensive genomic instability, pronounced heterogeneity, and complex regulatory interactions across multiple biological layers (Sayles et al., 2019; Zheng et al., 2024). Conventional statistical and hypothesis-driven approaches often struggle to extract robust biomarkers from high-dimensional omics data, particularly in the context of limited cohort sizes (Rahnenführer et al., 2023; Zong et al., 2024). These challenges have promoted the use of AI methods, including supervised learning for prediction, unsupervised learning for structure discovery, and deep learning for representation learning and multimodal integration. AI-driven frameworks enable a transition from single-marker discovery toward composite biomarker signatures that more accurately reflect disease biology (Cui et al., 2025; Nie et al., 2025). By integrating commonly used osteosarcoma omics layers such as bulk transcriptomics and, when available, epigenetic or single-cell profiles, these approaches can support prognostic stratification and candidate prioritization (Figure 1).

### 4.2 Machine learning approaches for biomarker identification

Machine learning (ML) techniques have been widely applied to identify osteosarcoma-associated biomarkers from transcriptomic and other omics datasets (Shi et al., 2022; Pan et al., 2026). Supervised learning methods are commonly used for feature selection and construction of prognostic or predictive signatures, effectively reducing dimensionality while retaining biologically informative variables associated with clinical outcomes (Zhao Q. et al., 2025; Chen et al., 2026). Unsupervised and semi-supervised approaches have also been employed to identify molecular subtypes and uncover latent structures within osteosarcoma cohorts, facilitating patient stratification and subtype-specific biomarker discovery (Jiang et al., 2022). However, performance is sensitive to cohort size, batch effects, preprocessing choices, and validation design, and models can overfit when these factors are not tightly controlled.

### 4.3 Multi-omics integration and network-based modeling

Beyond single-omics analyses, AI-based integration of multi-omics datasets enables characterization of cross-layer regulatory interactions in osteosarcoma. Integrative models combining transcriptomic, epigenomic, proteomic, and metabolic data provide a more comprehensive representation of tumor biology. Network-based approaches, in particular, facilitate identification of key regulatory nodes and functional modules that may serve as biomarkers or therapeutic targets. Compared with single-feature differential analysis, network models can incorporate interaction context and prioritize hubs or modules that better capture coordinated dysregulation, improving biological interpretability and portability across datasets (Ni et al., 2023; Li et al., 2022). Nonetheless, the limited availability of matched multi-omics datasets in osteosarcoma remains a major constraint.

### 4.4 Challenges and validation considerations in AI-Derived biomarkers

Despite their promise, AI-driven biomarker discovery approaches face important challenges (Ching et al., 2018). Small sample sizes, cohort heterogeneity, and batch effects can compromise model generalizability, while limited interpretability of complex models may hinder clinical acceptance (Tung et al., 2017). In addition, many AI-derived biomarkers are reported without sufficient experimental or clinical validation. To improve reproducibility and translation, studies should emphasize transparent reporting, standardized pipelines, interpretable model outputs where possible, and validation in independent cohorts before clinical claims are made. Close collaboration between computational researchers, clinicians, and laboratory specialists is essential to ensure translational feasibility.

## 5 AI-assisted drug target identification and drug sensitivity prediction

### 5.1 AI-based target prioritization beyond single-gene screening

Traditional target discovery in osteosarcoma has largely relied on single-gene perturbation or pathway-focused screening, which often fails to account for network-level regulation and tumor heterogeneity (Sadrkhanloo et al., 2023; Yu et al., 2023). AI-based approaches provide a complementary strategy by systematically prioritizing candidate therapeutic targets from large-scale omics datasets (Wang et al., 2024; Zhou et al., 2020). By integrating gene expression profiles, mutation patterns, and pathway activity scores, machine-learning models can rank potential targets according to their association with disease progression, metastatic potential, or treatment response (Zhao W. et al., 2025). Importantly, this prioritization shifts attention from isolated alterations toward regulatory hubs and coherent modules, which may reduce sensitivity to context-specific signals. This systems-level perspective improves the likelihood of identifying targets with broader biological impact and reduces the risk of selecting context-dependent or non-essential candidates.

### 5.2 Network and systems biology–guided target identification

Network-based AI frameworks have been increasingly applied to map molecular interactions and identify key regulatory nodes in osteosarcoma (Chen, 2017; Vaiyapuri et al., 2022). By constructing gene–gene, protein–protein, or signaling pathway networks, these approaches enable the identification of highly connected or influential components that may serve as effective therapeutic targets (Li et al., 2021; Liu et al., 2015). Integration of network topology with clinical outcome data further refines target selection by prioritizing nodes that are both biologically central and clinically relevant (Pan et al., 2018; Xu et al., 2021). Such systems biology–guided strategies are particularly valuable in osteosarcoma, where extensive genomic instability and pathway redundancy complicate single-target interventions (Corre et al., 2020). Relative to single-gene ranking, network-guided prioritization can highlight combinatorial vulnerabilities and reduce reliance on any one marker’s stability across cohorts. Targeting network hubs or combinatorial vulnerabilities identified through AI-driven network analysis may offer more durable therapeutic effects than traditional single-pathway inhibition.

### 5.3 AI-driven drug sensitivity prediction and drug repurposing

Beyond target identification, AI methodologies have been widely employed to predict drug sensitivity and resistance patterns in osteosarcoma (Dufau et al., 2019; Salimi et al., 2025). By learning from transcriptomic signatures and pharmacogenomic datasets, predictive models can estimate tumor response to specific agents and identify molecular features associated with drug resistance (Iorio et al., 2016; Buondonno et al., 2016; Zhang H. J. et al., 2025). In practice, these models are mainly used to generate and prioritize testable hypotheses rather than to directly guide clinical prescribing. AI has also facilitated drug repurposing by matching disease-specific molecular signatures with drug-induced expression profiles (Wang et al., 2018). This strategy enables the identification of candidate compounds that may reverse malignant phenotypes or enhance chemotherapy sensitivity, potentially accelerating therapeutic development by leveraging existing drugs with established safety profiles. However, *in silico* predictions remain dependent on reference data relevance and require careful experimental validation before translational interpretation.

### 5.4 Limitations and translational challenges of AI-Guided therapeutic strategies

Despite their potential, AI-assisted target discovery and drug prediction face major translational barriers (Meng et al., 2023; Qu et al., 2024). Osteosarcoma-specific pharmacogenomic datasets remain limited, and many models rely on heterogeneous cancer data or cell line systems that incompletely represent patient tumors (Trujillo-Paolillo et al., 2023; Thomas et al., 2011). Discrepancies between predicted and observed responses also reflect tumor microenvironment influences that are difficult to capture computationally. Translating AI outputs into clinically actionable strategies is further hindered by model complexity, limited benchmarking, and weak integration with validation pipelines. Addressing these issues requires closer coupling of computational prioritization with functional assays, clinically relevant models, and well-designed translational studies. Overall, AI-assisted approaches expand target discovery and drug-response prediction through systematic, data-driven prioritization, but their clinical value depends on rigorous validation and practical implementation. The subsequent section focuses on translational validation strategies that bridge computational predictions and clinical application.

## 6 Translational validation: from computational prediction to clinical testing

### 6.1 Experimental validation of AI-derived biomarkers and targets

Translational validation represents a critical step in bridging AI-driven predictions and clinical application in osteosarcoma (Liu J. et al., 2025; He et al., 2025; Cai et al., 2025). Computationally identified biomarkers and therapeutic targets require experimental confirmation to establish biological relevance and functional significance (Li et al., 2024; Kathiresan et al., 2024). *In vitro* assays using osteosarcoma cell lines are commonly employed to evaluate the effects of candidate genes or pathways on proliferation, apoptosis, migration, and drug sensitivity (Zeng et al., 2025; Ou et al., 2025). These experiments mainly provide functional support for AI-derived hypotheses and help filter false positives from purely data-driven analyses, but they do not by themselves establish clinical utility. Beyond functional assays, expression validation at the protein or transcript level is essential to confirm the detectability and stability of proposed biomarkers (González Díaz et al., 2022; Glover et al., 2017). Consistency between computational predictions and experimental observations strengthens confidence in candidate selection and informs downstream translational development.

### 6.2 Validation using patient-derived samples and clinical cohorts

Patient-derived specimens play a central role in assessing the clinical relevance of AI-identified biomarkers (Mowery et al., 2024; Dean et al., 2018). Tissue samples obtained from surgical resection or biopsy enable validation of expression patterns in real-world clinical contexts, while liquid biopsy samples support evaluation of minimally invasive biomarkers for disease monitoring (An et al., 2025; Nakano, 2022). Linking biomarker measures to metastatic status, chemotherapy response, and survival provides clinical association evidence that is distinct from *in vitro* functional validation. However, cohort-based validation faces several challenges, including limited sample availability, inter-center variability, and differences in sample processing protocols. These factors underscore the importance of standardized procedures, independent cohort validation, and transparent reporting to ensure reproducibility and generalizability.

### 6.3 Analytical standardization and clinical feasibility considerations

For AI-derived biomarkers to be clinically actionable, analytical performance and feasibility must be carefully evaluated (Sanders et al., 2004; Freeland et al., 2024). Assay sensitivity, specificity, reproducibility, and turnaround time are critical determinants of clinical utility, particularly in the context of osteosarcoma, where timely treatment decisions are essential (Kager et al., 2015; Abedi et al., 2024; Peng et al., 2025). From a laboratory medicine perspective, biomarkers that can be assessed using standardized, cost-effective platforms are more likely to be adopted in routine practice. Practical bottlenecks such as assay standardization, pre-analytical variability, and implementation constraints can delay translation even when biological signals are strong. In addition, the translation of computational models into clinically interpretable tools remains a key challenge. Simplifying complex multi-gene signatures into practical testing panels and ensuring compatibility with existing diagnostic workflows are necessary steps toward clinical implementation.

## 7 Discussion

Recent years have witnessed notable advances in the identification of biomarkers and therapeutic targets in osteosarcoma; however, effective clinical translation remains limited (Di Gregorio et al., 2024; Wang and Shi, 2025). Traditional experimental approaches have generated important mechanistic insights but are often constrained by their hypothesis-driven focus on isolated molecular events, which inadequately captures the extensive heterogeneity and network-level regulation characteristic of osteosarcoma (Kansara et al., 2014; Serra and Hattinger, 2017).

The integration of artificial intelligence–based methodologies offers a promising strategy to address these limitations (Zhang et al., 2024; Zhong et al., 2025). By enabling systematic analysis of high-dimensional omics data, AI-driven approaches facilitate the identification of composite biomarker signatures and regulatory hubs that more accurately reflect tumor biology (Ji et al., 2023). Rather than replacing conventional experimental research, AI complements traditional approaches by prioritizing candidates with higher biological relevance and translational potential.

At the same time, AI-derived results should be interpreted with attention to study design. Limited cohort sizes, data heterogeneity, and batch effects increase the risk of overfitting and restrict model generalizability, particularly in a rare malignancy such as osteosarcoma. In addition, limited interpretability of complex models may hinder clinical adoption if mechanistic relevance is insufficiently established through experimental validation.

From a translational perspective, bridging the gap between computational prediction and clinical implementation remains a central challenge (D'Anna et al., 2025). Many AI-identified biomarkers and therapeutic targets have yet to be validated in patient-derived samples or assessed within standardized diagnostic workflows. Analytical robustness, assay feasibility, and cost-effectiveness are critical determinants of clinical utility, underscoring the importance of close collaboration among computational scientists, laboratory medicine specialists, and clinicians.

Overall, these considerations highlight the need for integrative frameworks that combine traditional experimental approaches with AI-driven analytics. Such strategies offer a more comprehensive understanding of osteosarcoma biology, improve biomarker robustness, and enhance prioritization of therapeutically actionable targets. Continued progress will depend on high-quality multi-omics datasets, standardized validation pipelines, and multidisciplinary collaboration to translate computational insights into clinically meaningful advances for patients with osteosarcoma.
